# From #cervicalcancerscreening to #LEEP – Quality and accuracy of cervical cancer content on TikTok

**DOI:** 10.1016/j.gore.2025.102018

**Published:** 2025-12-30

**Authors:** Harriet Rothschild, Kelsey Keverline, Sara McKinney, Maureen Farrell, Minhazur Sarker

**Affiliations:** Department of Obstetrics, Gynecology, and Reproductive Sciences, University of California San Diego, La Jolla, CA, USA

**Keywords:** Cervical cancer screening, Social media

## Abstract

**Objectives:**

Many reproductive-aged individuals use social media platforms to gather medical advice or find community. TikTok is one of the fastest growing social media platforms and used by many reproductive-aged individuals.

**Methods:**

We evaluated the top 100 English-language videos on cervical cancer screening and dysplasia treatment for relevance to the hashtag and content quality and accuracy.

**Results:**

Among the included videos, most of the content highlighted patients’ personal experiences and provided little medical educational value. Notably, the videos created by medical professionals were higher quality and more often contained accurate health information.

**Conclusions:**

This study highlights the need to increase content quality on TikTok to raise awareness and uptake for cervical cancer screening and treatment in reproductive-aged individuals.

## Highlights

• The most viewed cervical cancer screening content on TikTok does not contain useful medical information.
• The supplements created by medical professionals were higher quality and more often contained accurate health information.
• Medical educational content on TikTok by health professionals provides an opportunity for improved outreach and engagement.

## 1 Introduction

Cervical cancer is the second most common cancer in reproductive-aged females worldwide both in terms of incidence and mortality (Wu et al., 2025). Timely screening and precancerous lesion treatment can prevent cervical cancer (Bouvard et al, 2021). The number of under-screened women in the US increased from 14.4 % in 2005 to 23.0 % in 2019 (Suk, R. et al., 2005) with disparities found across different sociodemographic groups, with lack of knowledge attributed as the biggest barrier (Suk, R. et al., 2005). Many patients report anxiety and fear surrounding pelvic exams and cervical cancer screening due to past experiences (O’Laughlin et al, 2021). Importantly, cervical cancer survivors often express a lack of reliable information on screening and prevention (Million, 2019).

Reproductive-aged individuals increasingly turn to and allow social media to supplement health information and influence their behaviors (Giacometti, et al., 2024, Kirkpatrick et al., 2024). One in five reproductive-aged individuals utilizes TikTok, a widely-used, video-based platform with 1.6 billion monthly users worldwide (Kirkpatrick et al., 2024, Singh, 2025). Given the apprehension surrounding cervical cancer screening despite the importance, we conducted an exploratory analysis of TikTok cervical cancer screening content.

## 2 Methods

A cross-sectional analysis of TikTok cervical cancer screening content was performed using four hashtags: #cervicalcancerscreening, #papsmear, #colposcopy, and #LEEP. These hashtags were chosen because we anticipated that they would encompass both broad and specific content. The Society of Gynecology Oncology (SGO) recommended the standardized hashtags #cervicalcancer and #papsmear in a 2021 Joint Statement (SGO, 2021). The top 100 English-language videos by view count were generated using the Apify TikTok Data Extractor on March 18th, 2025. The primary outcome was the number of relevant videos, defined as content discussing cervical cancer screening or treatment. Secondary outcomes included thematic categorization of content into pre-identified categories and Global Quality Score (GQS). GQS is a validated tool to evaluate medical information quality with a range from one, indicating content that was not useful, to five, indicating very useful content. Medical education content was evaluated using the total modified Discern Score (mDISCERN) – a validated tool to assess medical claims quality. The mDISCERN is a cumulative score with a maximum of five representing stronger content and zero representing the weakest content based on five separate domains (clear aims, reliable sources, balanced and unbiased sources, additional sources listed, and areas of uncertainty stated). A video was determined to make medical claims if the content was instructional, educational, or provided advice about medical procedure or topic.

Continuous variables are presented as median and interquartile range (IQR), and categorical variables as frequencies. All descriptive analyses were performed on STATA IC 15.1 (College Station, Texas, USA). The study was exempt from Institutional Review Board oversight since publicly available data was used.

## 3 Results

Among the top 100 English-language videos for #cervicalcancerscreening, #papsmear, #colposcopy, and #LEEP, 99, 93, 87 and 94, were relevant, respectively (Table 1). Most creators were women, based in the United States, and did not identify as healthcare providers (Table 1). Among the two generic hashtags, #cervicalcancerscreening and #papsmear, more videos were made by healthcare professionals and shared medical education or non-medical entertainment (Table 2). For the two more specific hashtags, #LEEP and #colposcopy, more videos represented patients’ personal experiences.

**Table 1:** **TikTok Creator Characteristics**: All categorical variables represented as n (%) and all continuous variables as median [interquartile range].

Videos Relevant to Hashtag	**#cervicalcancerscreening** **Total N = 100** Included n = 99	**#papsmear** **Total N = 100** Included n = 93	**#colposcopy** **Total N = 100** Included n = 87	**#LEEP** **Total N = 100** Included n = 94
Creator Follow Count, median [IQR]	42,050 [2600.5, 414650]	14,400 [1526, 494000]	43,200 [2690, 579400]	11,550 [965.5, 100550]
Creator Credentials				
Physician	25 (25.0)	16 (17.2)	10 (11.5)	11 (11.7)
Registered Nurse	6 (6.0)	5 (5.4)	3 (3.5)	3 (3.2)
Other Healthcare Provider*	19 (19.0)	15 (16.1)	10 (11.5)	7 (7.4)
Other	44 (44.0)	53 (57.0)	27 (31.0)	39 (41.5)
None Listed	6 (6.0)	4 (4.3)	37 (42.5)	34 (36.2)
Creator Gender				
Woman	86 (86.0)	84 (90.3)	79 (90.8)	89 (94.7)
Man	8 (8.0)	3 (3.2)	5 (5.8)	3 (3.2)
Transgender	2 (2.0)	1 (1.1)	0 (0.0)	0 (0.0)
Unsure	4 (4.0)	5 (5.4)	2 (2.3)	2 (2.1)
Creator in U.S.	66 (66.0)	79 (85.0)	66 (75.9)	60 (63.8)

*Includes advanced practice providers, midwives, doulas, and students in training.

**Table 2:** **TikTok Video Characteristics**: All categorical variables represented as n (%) and all continuous variables as median [interquartile range].

	**#cervicalcancerscreening** **Total N = 100** Included n = 99	**#papsmear** **Total N = 100** Included n = 93	**#colposcopy** **Total N = 100** Included n = 87	**#LEEP** **Total N = 100** Included n = 94
Number of Views	573,500 [279100, 1100000]	859,250 [388600, 2600000]	621,700 [202900, 2000000]	410,450 [141900, 1300000]
Number of Comments	107 [18.5, 310]	98 [18, 835]	68 [23, 323]	47 [17, 138]
Number of Shares	70.5 [13, 523]	183.5 [11, 1607]	31 [2, 259]	15 [3, 75]
Duration of Video (in seconds)	47 [15, 78.5]	28.5 [9, 62]	62 [15, 172]	97 [53, 180]
Response Video	17 (17.0)	8 (8.6)	17 (19.8)	21 (22.3)
Type of Video Content				
Entertainment	16 (16.0)	18 (19.4)	6 (6.9)	5 (5.3)
Medical Education	44 (44.0)	31 (33.3)	14 (16.1)	20 (21.3)
Patient Advocacy	11 (11.0)	6 (6.5)	8 (9.2)	2 (2.1)
Personal Experience	28 (28.0)	36 (38.7)	53 (60.9)	61 (64.9)
Lifestyle	1 (1.0)	2 (2.2)	2 (2.3)	3 (3.2)
Other	0 (0.0)	0 (0.0)	4 (4.6)	3 (3.2)
Encourages to Decline Intervention	1 (1.0)	23 (25.0)	0 (0.0)	2 (2.1)
Encourages to Distrust Healthcare	1 (1.0)	14 (15.1)	16 (18.4)	6 (6.4)
Fear Surrounding Pathology	5 (5.0)	35 (37.6)	31 (35.6)	16 (17.0)
Comments Support Content	74 (74.0)	73 (78.5)	66 (75.9)	71 (75.5)
Compelling Story	26 (26.0)	17 (18.5)	27 (31.0)	26 (28.0)
Visual Elements or Pictures	54 (54.0)	30 (32.3)	13 (14.9)	9 (9.6)
Music	35 (35.0)	34 (36.6)	37 (42.5)	22 (23.4)
Comedic Elements	22 (22.0)	28 (30.1)	17 (19.5)	10 (10.6)
Global Quality Score	3 [2,4]	2 [1,3]	2 [1,3]	2 [1,3]
1 = Not at all Useful	12 (12.0)	33 (35.5)	33 (37.9)	43 (45.7)
2 = Limited Use	17 (17.0)	20 (21.5)	25 (28.7)	18 (19.2)
3 = Somewhat Useful	27 (27.0)	20 (21.5)	19 (21.8)	20 (21.3)
4 = Useful	20 (20.0)	12 (12.9)	7 (8.1)	7 (7.5)
5 = Very Useful	24 (24.0)	8 (8.6)	3 (3.5)	6 (6.4)
Making Medical Claims	n = 68 (68.0)	n = 46 (49.5)	n = 37 (42.5)	n = 38 (40.4)
Inaccurate/Misleading	3 (4.4)	8 (17.4)	5 (13.5)	9 (23.7)
Partially Accurate	10 (14.7)	7 (15.2)	16 (43.2)	18 (47.4)
Accurate/Evidence Based	55 (55.0)	31 (67.4)	16 (43.2)	11 (29.0)
mDISCERN Score***	3 [1,3]	3 [1,3]	1 [1,3]	1 [0,3]

**Total Modified Discern Score is a cumulative score of five separate domains (clear aims, reliable sources, balanced and unbiased sources, additional sources listed, and areas of uncertainty stated; range from 0 to 5, higher score represents stronger content).

For #papsmear, 25 % of videos discussed declining interventions and for both #papsmear and #colposcopy, 15.1 % and 18.4 % of videos, respectively, discussed distrusting providers (Table 2). Similarly, #papsmear and #colposcopy had more themes of fear than other hashtags. The median [IQR] GQS was higher for #cervicalcancerscreening at 3 [2,4] than for all other hashtags at 2 [1,3]. While #cervicalcancerscreening and #papsmear had higher mDISCERN scores (median IQR 3 [1,3]), the scores were lower for #colposcopy and #LEEP at 1 [1,3] and 1 [0,3], respectively.

Of the 373 relevant videos analyzed, 189 (50.7 %) made medical claims and most were partially or completely evidence-based (Table 2). Across all hashtags, among 62 (16.6 %) videos made by physicians, the GQS was 4 [3,5] suggesting higher medical utility. Although most videos included supportive comment sections, some popular comments shared strong negative experiences with cervical cancer screening which may have been further boosted from algorithms prioritizing comments with increased engagement. The top-performing videos frequently incorporated catchy music alongside personal narratives. Additional video characteristics are noted in Table 1.

## 4 Conclusions

Our findings reveal that hashtags related to screening (#cervicalcancerscreening and #papsmear) contained more consistently accurate medical information than hashtags related to treatment (#colposcopy and #LEEP). This may be because hashtags related to screening were more frequently made by healthcare providers. Normalizing cervical cancer screening and treatment on social media may have psychological benefits and increase uptake. While content created by physicians had significantly higher quality, only a few of the top-performing content were created by physicians. This highlights the necessity for physicians to engage on TikTok and recognize TikTok as an information source for their patients during routine preventative healthcare visits.

Notably, this gap is reinforced by prior research demonstrating that reproductive-aged individuals express a preference for content delivered by qualified health professionals (Kirkpatrick et al., 2024). SGO does not have an official TikTok account, although it does publish content on other social media platforms. SGO along with the other major Gynecologic societies published a joint statement in 2021 to conform hashtags to help streamline social media communication for medical education across specialties, although implementation and content quality are varied (SGO, 2021).

The opportunity for national organizations such as the American College of Obstetricians and Gynecologists or the American Society for Colposcopy and Cervical Pathology to spearhead educational social media outreach is ripe. Physicians may be on social media for a variety of reasons including patient recruitment, creative outlet, or self-promotion, but the clinical value of this content is a component that may be underestimated. Whether more prominent engagement by healthcare professionals could help improve cervical cancer screening uptake and affect patient outcomes remains to be seen.

Increasing healthcare provider presence and medical education for cervical cancer screening and treatment on social media may be an important step to improving awareness and uptake in reproductive-aged individuals.

## CRediT authorship contribution statement

**Harriet Rothschild:** Writing – original draft, Data curation. **Kelsey Keverline:** Writing – review & editing, Data curation. **Sara McKinney:** Writing – review & editing, Supervision, Investigation, Conceptualization. **Maureen Farrell:** Writing – review & editing, Supervision, Methodology, Investigation, Conceptualization. **Minhazur Sarker:** Writing – review & editing, Supervision, Project administration, Methodology, Investigation, Formal analysis, Data curation, Conceptualization.

## Funding

Dr. Sarker’s time was supported by an NIH T32 (HD007203-42) grant.
